# Quantitative Amyloid Imaging in Autosomal Dominant Alzheimer’s Disease: Results from the DIAN Study Group

**DOI:** 10.1371/journal.pone.0152082

**Published:** 2016-03-24

**Authors:** Yi Su, Tyler M. Blazey, Christopher J. Owen, Jon J. Christensen, Karl Friedrichsen, Nelly Joseph-Mathurin, Qing Wang, Russ C. Hornbeck, Beau M. Ances, Abraham Z. Snyder, Lisa A. Cash, Robert A. Koeppe, William E. Klunk, Douglas Galasko, Adam M. Brickman, Eric McDade, John M. Ringman, Paul M. Thompson, Andrew J. Saykin, Bernardino Ghetti, Reisa A. Sperling, Keith A. Johnson, Stephen P. Salloway, Peter R. Schofield, Colin L. Masters, Victor L. Villemagne, Nick C. Fox, Stefan Förster, Kewei Chen, Eric M. Reiman, Chengjie Xiong, Daniel S. Marcus, Michael W. Weiner, John C. Morris, Randall J. Bateman, Tammie L. S. Benzinger, Dominantly Inherited Alzheimer Network

**Affiliations:** 1 Department of Radiology, Washington University School of Medicine, Saint Louis, Missouri, United States of America; 2 Department of Neurology, Washington University School of Medicine, Saint Louis, Missouri, United States of America; 3 Department of Radiology, University of Michigan, Ann Arbor, Michigan, United States of America; 4 University of Pittsburgh School of Medicine, Pittsburgh, Pennsylvania, United States of America; 5 University of California San Diego, La Jolla, California, United States of America; 6 Columbia University, New York, New York, United States of America; 7 Department of Neurology, Keck School of Medicine, University of Southern California, Los Angeles, California, United States of America; 8 Keck School of Medicine, University of Southern California, Los Angeles, California, United States of America; 9 Department of Radiology, Indiana University, Indianapolis, Indiana, United States of America; 10 Massachusetts General Hospital, Harvard Medical School, Boston, Massachusetts, United States of America; 11 Butler Hospital and Brown University, Providence, Rhode Island, United States of America; 12 Neuroscience Research Australia and School of Medical Sciences, University of New South Wales, Sydney, New South Wales, Australia; 13 The Florey Institute and the University of Melbourne, Parkville, Victoria, Australia; 14 Dememtia Research Centre, Institute of Neurology, London, Great Britain; 15 Deutsches Zentrum für Neurodegenerative Erkrankungen (DZNE) München/Tübingen and Dept. of Nuclear Medicine, Technische Universität München, München, Germany; 16 Banner Alzheimer’s Institute, Banner Health, 901 E. Willetta Street, Phoenix, Arizona, United States of America; 17 Division of Biostatistics, Washington University School of Medicine, Saint Louis, Missouri, United States of America; 18 Department of Radiology, University of California San Francisco, San Francisco, California, United States of America; University of Manchester, UNITED KINGDOM

## Abstract

Amyloid imaging plays an important role in the research and diagnosis of dementing disorders. Substantial variation in quantitative methods to measure brain amyloid burden exists in the field. The aim of this work is to investigate the impact of methodological variations to the quantification of amyloid burden using data from the Dominantly Inherited Alzheimer’s Network (DIAN), an autosomal dominant Alzheimer’s disease population. Cross-sectional and longitudinal [^11^C]-Pittsburgh Compound B (PiB) PET imaging data from the DIAN study were analyzed. Four candidate reference regions were investigated for estimation of brain amyloid burden. A regional spread function based technique was also investigated for the correction of partial volume effects. Cerebellar cortex, brain-stem, and white matter regions all had stable tracer retention during the course of disease. Partial volume correction consistently improves sensitivity to group differences and longitudinal changes over time. White matter referencing improved statistical power in the detecting longitudinal changes in relative tracer retention; however, the reason for this improvement is unclear and requires further investigation. Full dynamic acquisition and kinetic modeling improved statistical power although it may add cost and time. Several technical variations to amyloid burden quantification were examined in this study. Partial volume correction emerged as the strategy that most consistently improved statistical power for the detection of both longitudinal changes and across-group differences. For the autosomal dominant Alzheimer’s disease population with PiB imaging, utilizing brainstem as a reference region with partial volume correction may be optimal for current interventional trials. Further investigation of technical issues in quantitative amyloid imaging in different study populations using different amyloid imaging tracers is warranted.

## Introduction

Alzheimer’s disease (AD) is the most common form of dementia [1] with its prevalence expected to dramatically increase in the next 50 years [2]. AD pathology begins to accumulate at least 10 to 20 years before clinical symptoms appear [1,3,4], and there is a growing consensus that effective treatment of AD will require early intervention [5,6]. The amyloid cascade is the primary target of the largest ongoing clinical trials [7], including DIAN-TU (DIAN Trial Unit) [8], A4 trial (Anti-Amyloid Treatment in Asymptomatic Alzheimer’s Disease) [9], and API (Alzheimer’s Prevention Initiative) [10] in preclinical population. Well validated biomarkers of amyloid accumulation are needed for these treatment development efforts and trial design [5,6].

The most commonly used quantification technique for amyloid Positron Emission Tomography (PET) imaging involves comparing regional uptake to cerebellar cortex [11,12]. However, the observation of amyloid deposition in the cerebellar cortex of familial AD cases [13,14] had led to the identification and validation of pons as an alternative reference region [15]. A large white matter region of interest (ROI) has been proposed as an reference region to provide improved discrimination between clinically defined groups [16]; and a smaller “core” white matter reference region was reported to improve the detection of changes in tracer retention in longitudinal studies within the Alzheimer’s Disease Neuroimaging Initiative (ADNI) [^18^F]-florbetapir data [17].

Correction of partial volume effects represents another area of active investigation [18]. PET imaging based measurement is subjected to partial volume effects, because of the low spatial resolution [19]. Although partial volume effects are well-recognized, correction techniques remain controversial. One study comparing two-component (brain and non-brain) vs. three-component (gray matter, white matter, non-brain) reported relative advantages and disadvantages of both methods [20]. Thomas *et al*. [21] reported improved quantitative accuracy using a region-based voxel-wise partial volume correction (PVC) method. More recently, Brendel *et al*. [16] and our group [18] found PVC improved the power to detect longitudinal amyloid burden change.

A third methodological choice concerns the quantitative model used to determine amyloid burden. The most commonly used approach is the target-to-reference region standard-uptake-value-ratio (SUVR) [4,16,17]. SUVR evaluation is simple and only requires a short acquisition, which translates to low cost. However, SUVR measurements are sensitive to the choice of temporal window used for evaluation, in part, owing to individual variability in cerebral perfusion [16,22]. Alternatively, kinetic modeling techniques [23,24] can be used to calculate distribution volume ratio (DVR) or binding potential (BP_ND_) as a quantitative measure of amyloid burden [11,22]. These techniques require longer dynamic PET acquisitions initiated in synchrony with tracer administration and are less attractive because of increased participant burden, study cost and complexity of quantification.

It should be pointed out that currently a “gold standard” technique that objectively measures brain amyloid burden is not available for validation of in vivo amyloid PET measurements. Some other criteria are often used to judge the performance of amyloid quantification techniques. These criteria include low inter-subject variability in reference regions [15,22], strong group differences between control subjects and AD patients [15,16,22], and, in longitudinal studies, strong longitudinal changes [16,17,25]. Here, we investigate the impact of 1) reference region selection; 2) correction for partial volume effects; and 3) choice of quantification technique using cross-sectional and longitudinal [^11^C]-Pittsburgh Compound B (PiB) imaging data from the DIAN study. Regional SUV and volume of distribution (V_T_) were used to determine the stability of reference regions tracer uptake by comparing mutation carriers against non-carriers. The power of detecting longitudinal changes was also examined to compare different amyloid quantification techniques.

## Materials and Methods

### Participants

The participants included in this study were recruited as part of the international Dominantly Inherited Alzheimer Network (DIAN) [26]. The DIAN includes individuals from families with known autosomal dominant mutation in amyloid precursor protein [27], presenilin 1 [28], or presenilin 2 [29] genes leading to early onset AD. The current study was based on the eighth semiannual data cutoff with a total of 341 participants who had at least one PiB scan; among them 203 were mutation carriers (APP = 27, PSEN1 = 161, PSEN2 = 15); within these 203 participants, 59 had usable PiB scans for at least two visits on the same scanners and formed the longitudinal mutation carrier cohort (LC). For participants with more than two PiB scans, the first two usable visits with an interval as close to 2 years as possible (range 0.8–3.3 yrs) was included in this study. The same scanner rule was enforced to minimize the impact of scanner difference. Within the LC cohort, a subset of 23 participants (LC_Dyn) had full dynamic scans at both time points. A longitudinal cohort for non-carriers (LNC) was also selected using the same criteria and had a total of 36 participants. A cross-sectional cohort (CC) was selected from the Washington University in St. Louis (WU) site DIAN cohort, including 69 participants (38 mutation carriers) with valid baseline PiB scans on the same PET scanner. Single scanner data minimizes differences in scanner calibration protocols. In addition, 42 participants (21 mutation carriers) from the WU site who had time-of-flight (TOF) MR angiography (MRA) scans and 70-min dynamic PiB PET scans were included for absolute quantification (AQ) of PiB binding using an image-derived arterial input function (IDAIF) technique [30]. Demographic details of these cohorts are provided in Table 1.

**Table 1:** Demographics Summary.

Cohort	LC	LC_Dyn	CC_Carrier	CC_Noncarrier	AQ_Carrier	AQ_Noncarrier	LNC
N	59	23	38	31	21	20	36
Age (SD) years	42.6 (8.7)	44.8 (10.8)	38.4 (10.5)	39.3 (9.5)	37.4 (11.2)	39.9 (9.1)	41.3 (9.6)
EYO (SD) years	-2.1 (8.6)	-1.1 (11.3)	-8.8 (9.4)	-5.7 (10.9)	-10.0 (10.3)	-5.5 (9.6)	-4.5 (10.2)
Education (SD) years	14.0 (2.8)	15.0 (3.2)	15.5 (3.1)	15.5 (2.4)	15.2 (2.6)	15.5 (2.5)	14.9 (2.2)
Interval (SD) years	1.7 (0.8)	1.9 (0.8)	-	-	-	-	2.4 (0.9)
Male (%)	30 (50.8)	17 (73.9)	27 (71.1)	15 (48.4)	16 (76.1)	9 (45.0)	14 (38.9)
APOE4+ (%)	23 (39.0)	11 (47.8)	14 (36.8)	10 (32.3)	11 (52.3)	5 (25.0)	11 (30.6)
CDR>0 (%)	28 (47.5)	10 (43.5)	11 (28.9)*	0 (0)	4 (19.0)	0 (0)	0 (0)
MMSE (SD)	26.2 (5.2)	28.2 (2.5)†	28.2 (3.4)*	29.6 (0.6)	28.1 (3.1)	29.4 (0.7)	29.1 (1.1)

LC = longitudinal cohort; CC_Carrier = cross-sectional cohort mutation carrier; CC_Noncarrier = cross-sectional cohort non-carrier; AQ_Carrier = absolute quantification cohort mutation carrier; AQ_Noncarrier = absolute quantification cohort non-carrier; LNC = longitudinal non-carrier cochort; baseline values were reported for LC and LNC; EYO = estimated years to onset; SD = standard deviation; APOE4+ = apolipoprotein E ε4 gene carrier; CDR = clinical dementia rating; MMSE = Mini Mental State Examination

*significantly different from corresponding non-carrier group

^†^significantly different from the LC group

All assessment and imaging procedures were approved by the WU Human Research Protection Office. Written informed consent was obtained from all individuals or their care-givers. Local institutional review boards (Columbia University Institutional Review Board; University of Pittsburgh Human Research Protection Office; UCLA Institutional Review Boards; Indiana University Institutional Review Boards; Partners Human Research Committee; Butler Hospital's Institutional Review Board for Human Subjects Research; The University of New South Wales Human Research Ethics Committee; University of Melbourne Office for Research Ethics and Integrity; Edith Cowan University Human Research Ethics Committee; and University College London Research Ethics Committee) also approved the collection of scans for archiving and future study at each non-WU study site.

### Clinical Assessment

The clinical assessment protocol has been previously described [3,4]. In brief, dementia status was assessed using the clinical dementia rating (CDR) [31]. Estimated year to onset (EYO) was calculated as the difference between the participant’s age at evaluation and the age at which parental cognitive decline began [3]. The ADAD mutation status was determined using polymerase chain reaction based amplification of the appropriate exon followed by Sanger sequencing [3]. Each participant’s apolipoprotein E (*APOE*) genotype was determined using previously described methods [32]. Clinical evaluators were blind to participant mutation status.

### Image Acquisition

Imaging protocol has been described in detail previously [4]. PiB PET acquisition consisted of either a 70-min dynamic scan starting at injection or a 30-min scan with 40 minutes uptake time. Accelerated 3D sagittal T1-weighted images of the head were acquired in each participant. TOF-MRA data was acquired with during the same imaging session as the T1-weighted structural scan for the AQ cohort.

### Image Analysis

Our image analysis technique has been previously described in detail [12]. In summary, FreeSurfer v5.1 (Martinos Center for Biomedical Imaging, Charlestown, Massachusetts, USA, https://surfer.nmr.mgh.harvard.edu/fswiki) was used to automatically segment the brain using T1-weighted MR acquired in corresponding visit as the PET scan. A PET Unified Pipeline (PUP) (https://github.com/ysu001/PUP) was used for automated PET data analysis [12,18]. In the default SUVR analysis, regional SUVR was calculated using cerebellar cortex as the reference region and PET data acquired between 40 and 70 minutes post-injection. When a full 70-min dynamic scan was available, regional time-activity curve for each ROI was extracted followed by regional binding potential (BP_ND_) (DVR-1) estimation using Logan graphical analysis with the cerebellar cortex (CER) serving as the reference [23]. Mean cortical binding potentials (MCBP) [11] and mean cortical SUVR (MCSUVR) were calculated based on a selected set of cortical FreeSurfer regions [12]. PVC was also performed using a regional spread function (RSF) technique implemented in PUP [18], which is also known as the geometric transfer matrix (GTM) technique [33]. Regional SUVR and BP_ND_ were estimated with and without PVC.

### Alternative Reference Region

**Fig 1:**
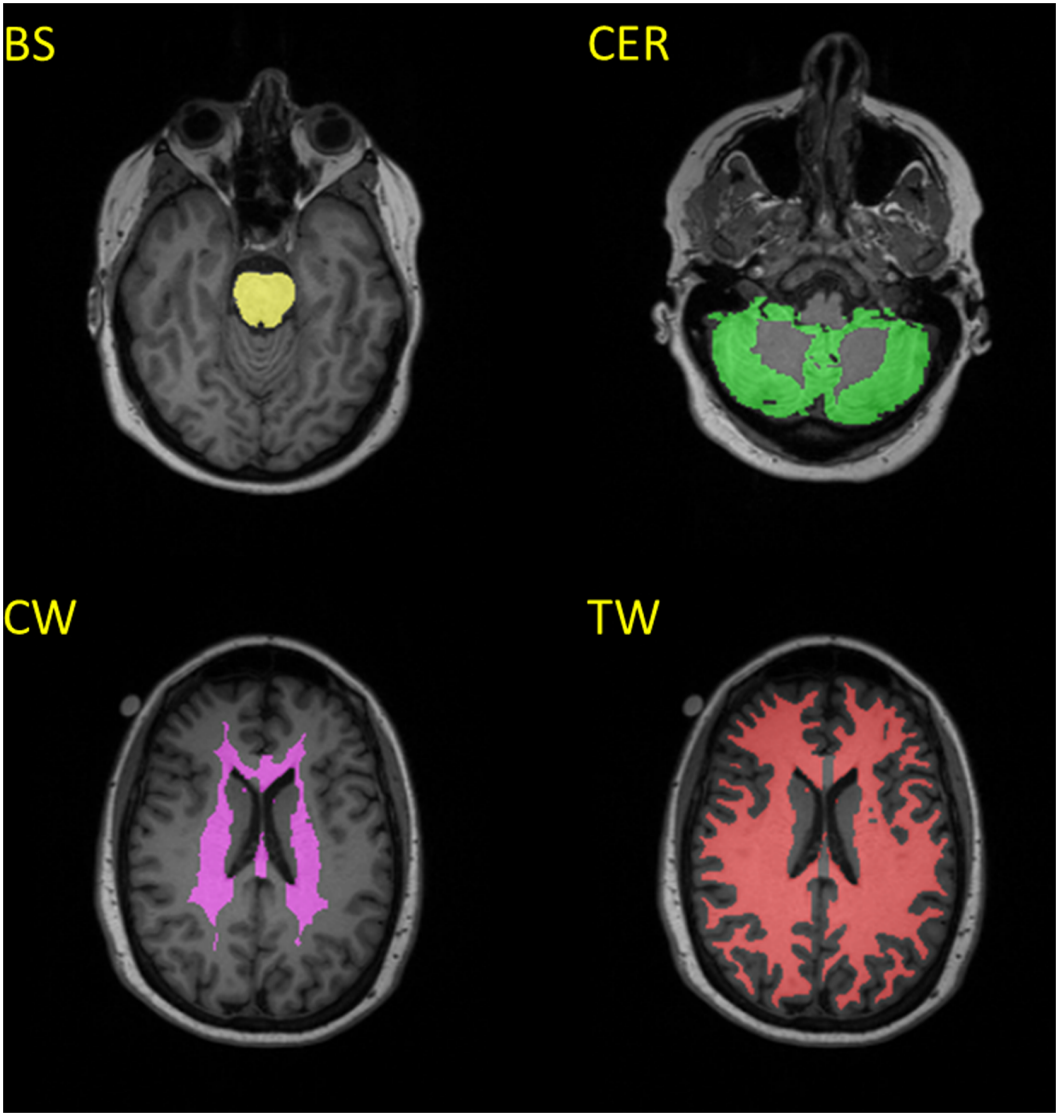
Example reference regions. (BS: Brain-Stem; CER: Cerebellar Cortex; CW: Core White Matter; TW: Total White Matter).

Recent papers [15–17] have proposed three alternative SUVR reference regions. We implemented these alternative reference regions based on FreeSurfer segmentation (Fig 1). The brain stem (BS) region was used to approximate the pontine reference, as proposed by Edison *et al*. [15]. A core white matter (CW) reference region, as proposed by Chen et al. [17], was approximated by combining corpus callosum and the ROI labelled as “UnsegmentedWhiteMatter” by FreeSurfer. A total white matter (TW) reference region was also constructed using all the cerebral white matter [16].

### Cross-sectional SUV Analysis

For the cross-sectional cohort, regional SUV were estimated by normalizing regional activity concentration with injection dose and body weight. SUV values were calculated for the four candidate reference regions (CER, BS, CW, and TW) and the mean cortical (MC) regions using the same time window as the SUVR analysis.

### Absolute Quantification

An IDAIF was obtained using a recently developed technique by combined analysis of TOF-MRA, T1-weighted MR, and dynamic PET imaging data [30,34]. Logan graphical analysis [35] was then applied to obtain regional V_T_, which is a measurement of absolute tracer binding. The quantification was performed on the same set of regions as the SUV analysis, and results were presented with and without PVC.

### Longitudinal Analysis

Longitudinal change in amyloid burden was assessed as the absolute change (*delta*), percent change (*delta*%) and annual rate of change (*rate*) in MCSUVR using all four candidate reference regions with and without RSF PVC. Follow-up MCSUVRs were compared with their corresponding baseline measurements using paired t-test. Cohen’s d effect size measure was calculated based on the mean and standard deviation of the annual rate of change in MCSUVR. Percent change in MCSUVR was also compared between quantification methods using paired t-test. In addition, we also estimated the number of participants per arm needed to detect a 25% and 50% reduction in amyloid accumulation rate due to treatment with 80% power and a two-tailed type-I error of p = 0.05 in a 12-month placebo-controlled randomized clinical trial. The 25% reduction was selected to match a similar study [17]. The 50% reduction was included to approximate hypothesized treatment effects in anti-amyloid treatment trials. The sample size calculation was performed using MATLAB (R2015b) (The Mathworks Inc, Natick, MA) function: sampsizepwr, in the statistics and machine learning toolbox v10.1. The calculation was performed based on the observed annual rate of change in MCSUVR and its standard deviation. LNC cohort data were also analyzed as a test-retest study, since amyloid burden and its change are not expected in this cohort of young participants. A test-retest variability measure was defined as:*TRT*% = (|*M*_1_-*M*_2_|/*M*_1_)×100%, where *M*_*1*_ was the amyloid burden measurement at baseline and *M*_*2*_ was the follow-up measurement. The mean and standard deviation of *TRT*% was evaluated for the LNC cohort and compared among different quantification methods.

## Results

### Cross-sectional SUV Analysis

Significant MCSUV differences were observed between mutation carriers and non-carriers with and without PVC (Table 2). SUVs for the TW region were significantly different between mutation carriers and non-carriers, however, PVC removes this difference. Regional SUVs were not significantly different for any of the other three reference regions regardless of PVC.

**Table 2:** Regional SUV for the cross-sectional cohort.

	MC	CER	BS	CW	TW	MCRSF	CERRSF	BSRSF	CWRSF	TWRSF
**Noncarrier**	0.64±0.11	0.60±0.10	0.96±0.20	0.99±0.21	0.87±0.18	0.56±0.10	0.56±0.09	1.06±0.23	1.07±0.24	1.04±0.24
**Carrier**	0.97±0.39	0.62±0.12	0.97±0.20	1.00±0.20	1.00±0.27	1.26±0.73	0.58±0.12	1.07±0.24	1.02±0.20	1.01±0.21
**p**	9.10E-06	3.55E-01	8.53E-01	9.29E-01	1.43E-02	8.76E-07	3.37E-01	8.92E-01	3.27E-01	6.60E-01

MC = mean cortical regions; CER = cerebellar cortex; BS = brainstem; CW = core white matter; TW = total white matter; MCRSF = mean cortical regions with RSF partial volume correction; CERRSF = cerebellar cortex with RSF partial volume correction; BSRSF = brainstem with RSF partial volume correction; CWRSF = core white matter with RSF partial volume correction; TWRSF = total white matter with RSF partial volume correction; p is the strength of the group difference, i.e. noncarrier vs. carrier, for each region of interest based on student t-test.

### Absolute Quantification

V_T_ was significantly different for MC regions between the mutation carriers and non-carriers, and PVC led to smaller p values (Table 3). No group differences in regional V_T_ were observed for any of the reference regions.

**Table 3:** Regional volume of distribution (VT) estimated using image-derived arterial input function.

	MC	CER	BS	CW	TW	MCRSF	CERRSF	BSRSF	CWRSF	TWRSF
Noncarrier	2.79±1.00	2.75±0.98	3.66±1.27	3.71±1.49	3.35±1.26	3.16±1.14	2.83±1.02	4.20±1.45	4.13±1.81	3.90±1.56
Carrier	3.70±1.07	2.71±0.62	3.55±0.80	3.68±0.86	3.77±0.87	5.20±2.06	2.80±0.64	4.06±0.93	3.79±1.01	3.72±0.86
p	7.78E-03	8.83E-01	7.28E-01	9.22E-01	2.27E-01	4.17E-04	9.07E-01	7.14E-01	4.69E-01	6.54E-01

MC = mean cortical regions; CER = cerebellar cortex; BS = brainstem; CW = core white matter; TW = total white matter; MCRSF = mean cortical regions with RSF partial volume correction; CERRSF = cerebellar cortex with RSF partial volume correction; BSRSF = brainstem with RSF partial volume correction; CWRSF = core white matter with RSF partial volume correction; TWRSF = total white matter with RSF partial volume correction; p is the strength of the group difference, i.e. noncarrier vs. carrier, for each region of interest based on student t-test.

### Longitudinal Analysis

For the LC cohort, using either cerebellar cortex or brain stem as the reference region, longitudinal MCSUVR change was not significant without partial volume, while PVC revealed the change (Table 4). Using core white matter and total white matter reference regions revealed significant longitudinal change in MCSUVR with or without PVC (Table 4). The MCSUVR change from baseline to follow-up had the smallest p values with (p = 0.0000075) and without (p = 0.000017) PVC when core white matter was used as the reference. PVC and using white matter as reference region also led to larger effect size (Table 4). PVC based analysis generated significantly (p<0.0005) larger longitudinal percent change in MCSUVR regardless of the reference regions used (Table 4). PVC and adoption of white matter as reference led to a considerably smaller estimated sample size needed to achieve an 80% power and two-sided type-I error of p = 0.05 to detect a reduced rate of amyloid accumulation in hypothetical anti-amyloid therapy trials.

**Table 4:** Longitudinal SUVR analysis for mean cortical regions in mutation carriers.

	MC_CER	MC_BS	MC_CW	MC_TW	MCRSF_CER	MCRSF_BS	MCRSF_CW	MCRSF_TW
**Baseline**	1.73±0.58	1.15±0.35	1.08±0.31	0.99±0.14	2.54±1.28	1.43±0.63	1.53±0.73	1.43±0.56
**follow-up**	1.76±0.60	1.17±0.35	1.12±0.32	1.00±0.14	2.65±1.36	1.48±0.64	1.64±0.80	1.49±0.57
**delta**	0.03±0.11	0.01±0.07	0.04±0.07	0.01±0.03	0.11±0.26	0.05±0.10	0.11±0.17	0.06±0.11
**delta%**	1.63±7.04	1.51±6.30†	3.34±5.88	1.29±3.54	4.19±11.07*‡	4.08±9.86*‡	7.03±11.27*	5.05±9.82*
**p (follow-up vs. Baseline)**	7.22E-02	1.39E-01	1.70E-05	8.12E-03	8.78E-04	6.25E-04	7.46E-06	1.14E-04
**Rate**	0.01±0.08	0.00±0.04	0.02±0.04	0.01±0.02	0.07±0.18	0.02±0.06	0.07±0.13	0.04±0.07
**Effect Size**	0.15	0.07	0.55	0.38	0.38	0.38	0.51	0.50
**sample size (25% reduction in Rate)**	5714	24165	411	885	858	852	480	502
**sample size (50% reduction in Rate)**	1430	6043	105	223	216	215	122	127

MC_CER = mean cortical region SUVR using cerebellar cortex as reference; MC_BS = mean cortical region SUVR using brainstem as reference; MC_CW = mean cortical region SUVR using core white matter as reference; MC_TW = mean cortical region SUVR using total white matter as reference; MCRSF_CER = mean cortical region SUVR using cerebellar cortex as reference with RSF partial volume correction; MCRSF_BS = mean cortical region SUVR using brainstem as reference with RSF partial volume correction; MCRSF_CW = mean cortical region SUVR using core white matter as reference with RSF partial volume correction; MCRSF_TW = mean cortical region SUVR using total white matter as reference with RSF partial volume correction; delta = change in SUVR from baseline to follow-up; delta% = percent change in SUVR from baseline to follow-up; p is the strength of the difference between follow-up and baseline SUVRs based on a paired t-test; Rate = the annual rate of SUVR change; sample size is the estimated number of participants per arm needed to detect a 25% or a 50% reduction in amyloid accumulation rate due to treatment with 80% power and a two-tailed type-I error of p = 0.05 in a 12-month placebo-controlled randomized clinical trial.

*percent change in MCSUVR significantly greater with PVC than without (p<0.0005)

^†^percent change in MCSUVR significantly smaller than CW referencing (p<0.01)

^‡^percent change in MCSUVR with PVC significantly smaller than CW referencing (p<0.05)

For the LNC cohort, no change in MCSUVR was observed, as expected, between baseline and follow-up regardless of the quantification methods used. It is observed that cerebellar cortex referencing led to the smallest test-retest variability (Table 5), while the other reference regions did not differ significantly.

**Table 5:** Reproducibility of PiB measurements based on the longitudinal non-carriers cohort (LNC).

	MC_CER	MC_BS	MC_CW	MC_TW	MCRSF_CER	MCRSF_BS	MCRSF_CW	MCRSF_TW
**TRT% (Mean±SD)**	3.0±2.7	4.0±3.8	4.5±4.0	3.0±2.4	5.0±3.7	7.1±7.3	9.2±8.3	8.5±7.5

MC_CER = mean cortical region SUVR using cerebellar cortex as reference; MC_BS = mean cortical region SUVR using brainstem as reference; MC_CW = mean cortical region SUVR using core white matter as reference; MC_TW = mean cortical region SUVR using total white matter as reference; MCRSF_CER = mean cortical region SUVR using cerebellar cortex as reference with RSF partial volume correction; MCRSF_BS = mean cortical region SUVR using brainstem as reference with RSF partial volume correction; MCRSF_CW = mean cortical region SUVR using core white matter as reference with RSF partial volume correction; MCRSF_TW = mean cortical region SUVR using total white matter as reference with RSF partial volume correction; *TRT*% = (|*M*_1_-*M*_2_|/*M*_1_)×100% is the test-retest reproducibility of SUVR measurement.

### Impact of Dynamic Acquisition

The estimated sample sizes for anti-amyloid therapy trials were smaller based on LC_Dyn data compared to the LC and the observed longitudinal MCSUVR change had smaller p values and larger effect size (Table 6 vs. Table 4). PVC had less of an impact on BP_ND_ compared to SUVR analyses.

**Table 6:** Mean cortical measurement for longitudinal cohort participants with full dynamic PiB.

	MC_CER	MC_BS	MC_CW	MC_TW	MCRSF_CER	MCRSF_BS	MCRSF_CW	MCRSF_TW	MCBP	MCBPRSF
**Baseline**	1.83±0.59	1.18±0.35	1.12±0.31	1.02±0.16	2.80±1.33	1.50±0.64	1.60±0.71	1.54±0.62	0.62±0.45	1.33±0.93
**follow-up**	1.89±0.58	1.22±0.35	1.16±0.32	1.03±0.15	2.93±1.33	1.58±0.66	1.71±0.75	1.60±0.62	0.67±0.45	1.43±0.95
**Delta**	0.05±0.13	0.04±0.06	0.04±0.05	0.01±0.04	0.14±0.29	0.09±0.09	0.11±0.12	0.06±0.11	0.05±0.10	0.10±0.22
**p (follow-up vs. Baseline)**	6.34E-02	1.98E-03	4.47E-04	8.39E-02	3.33E-02	2.02E-04	1.68E-04	1.19E-02	2.91E-02	3.84E-02
**Rate**	0.02±0.09	0.02±0.03	0.02±0.03	0.00±0.02	0.05±0.17	0.04±0.05	0.05±0.06	0.03±0.05	0.02±0.05	0.04±0.11
**Effect Size**	0.23	0.62	0.71	0.25	0.33	0.85	0.82	0.49	0.33	0.34
**sample size (25% reduction in Rate)**	2286	333	251	2038	1171	177	188	519	1177	1097
**sample size (50% reduction in Rate)**	573	85	65	511	295	46	49	132	296	276

MC_CER = mean cortical region SUVR using cerebellar cortex as reference; MC_BS = mean cortical region SUVR using brainstem as reference; MC_CW = mean cortical region SUVR using core white matter as reference; MC_TW = mean cortical region SUVR using total white matter as reference; MCRSF_CER = mean cortical region SUVR using cerebellar cortex as reference with RSF partial volume correction; MCRSF_BS = mean cortical region SUVR using brainstem as reference with RSF partial volume correction; MCRSF_CW = mean cortical region SUVR using core white matter as reference with RSF partial volume correction; MCRSF_TW = mean cortical region SUVR using total white matter as reference with RSF partial volume correction; MCBP = mean cortical binding potential; MCBPRSF = mean cortical binding potential with RSF partial volume correction; delta = change in SUVR from baseline to follow-up; p is the strength of the difference between follow-up and baseline SUVRs based on a paired t-test; Rate = the annual rate of SUVR change; sample size is the estimated number of participants per arm needed to detect a 25% or a 50% reduction in amyloid accumulation rate due to treatment with 80% power and a two-tailed type-I error of p = 0.05 in a 12-month placebo-controlled randomized clinical trial.

## Discussion

### Reference Region

Both SUV analysis and IDAIF based absolute quantification showed all four reference regions were not different between mutation carriers and non-carriers. White matter referencing, especially using core white matter ROI, improved the sensitivity for detecting longitudinal changes in MCSUVR, however, the cause of this improvement is unclear and requires further investigation. White matter PiB uptake was not more stable in either the non-carrier control population or the mutation carrier participants. In fact, the coefficient of variation was greater for white matter than for cerebellar cortex and brain stem. In the LNC cohort, larger test-retest variability was observed when core white matter was used as reference region in comparison to cerebellar cortex referencing. On the other hand, a (non-significant) trend was observed towards lower white matter uptake (for both core and total white matter regions) for mutation carriers than noncarriers in the IDAIF analysis and partial volume corrected SUV, suggesting potential changes in white matter region properties in the mutation carriers that may alter white matter tracer uptake. We suspect such changes in the white matter region over time may, in part, be the cause of the improved power in detecting longitudinal MCSUVR change using white matter referencing. In addition, the observed longitudinal change in MCSUVR agreed well (Pearson r = 0.66, p = 1.03E-8) when cerebellar cortex and brain stem were used as the reference region. However, the longitudinal MCSUVR changes measured using white matter as reference region did not agree (Pearson r = 0.25, p = 0.052) with the results obtained using cerebellar cortex referencing. These differences suggest that using white matter as the reference region potentially leads to less consistent results.

### Partial Volume Correction

We show that PVC enhances sensitivity for the detection of longitudinal changes in PiB binding in the DIAN cohort, in accordance with our previous report in a sporadic AD cohort [18]. In fact, PVC consistently increased the observed longitudinal percent change in measured PiB uptake regardless of the reference region. Two separate characteristics of PVC technique may be the causes of the beneficial effects. Firstly, PVC amplifies the signal we want to detect more than the amplification of noise therefore improve signal to noise ratio. Secondly, without PVC, MCSUVR and its change over time is dependent upon both amyloid deposition and brain atrophy; when PVC is performed we can separate the two processes. Therefore, we strongly recommend PVC especially in longitudinal studies aimed at detecting changes in amyloid burden.

### Full Dynamic Acquisition and Modeling

Moderate improvement was observed when full dynamic PET acquisition was available. As previously reported [36], SUVR analysis is more sensitive to the choice of time window used for quantification. Improved statistical power with full dynamic scanning likely is attributable to greater consistency in the selection of the time window used for quantification. In short acquisitions, uptake time may vary from one scan to the next. Regional binding potential estimation provided improved statistical power in comparison to SUVR, as previously reported [36]. Nevertheless, full kinetic modeling requires longer scan time and the quantification requires more PET imaging expertise, both of which could lead to increased cost. However, such increases in cost may be offset by improved accuracy, increased statistical power and reduced sample size. This trade-off should be carefully evaluated at study design.

### Observed Longitudinal Change

In this study, moderate annual rate of change in amyloid burden was observed with substantial inter-individual variability. And it appears that the inter-individual variability was larger when cerebellar cortex was used as reference than when white matter were used as reference. Partial volume correction increased the magnitude of the annual rate of change no matter what reference region was used. Similar annual rate of change was observed in this cohort (0.02±0.05 in MCBP) in comparison to our previous observations in a sporadic AD cohort (0.016±0.03 in MCBP)[18]. Using ADNI [^18^F]-florbetapir data, Chen et al. [17] reported an annual rate of SUVR change of 0.013±0.011 in the asymptomatic amyloid positive group, and an annual rate of 0.012±0.014 in the MCI amyloid positive group using cerebral white matter as the reference. A similar rate of change was also reported by Brendel et al. [16] using white matter as reference region based on the same dataset. In comparison, in this current study in the DIAN cohort using PiB as the tracer, we observed a substantially larger annual rate of 0.02±0.03 in SUVR in a cohort that is a mixture of MCI (CDR = 0.5) and asymptomatic participants using similar reference region. The estimated sample size needed for anti-amyloid trials were similar between the two studies. The difference in annual rate of change and similarity of estimated sample size is likely a combined effects of cohorts, tracer, and quantification methods, and further investigation is necessary.

### Limitations

The analysis performed in this study is based on the DIAN cohort using PiB as the imaging tracer. Whether our observations can be translated to other cohorts and/or using other amyloid tracers remains to be determined. We focused our comparison of quantification methodology on those utilizing structural MR obtained on the same participant. While MR data is generally available for most of the ongoing neuroimaging studies, quantification methods that does not require MR [37–39] have their advantages and warrant further investigation. In this study, we focused on a single PVC technique in based on our previous experience [18]. This technique depends on segmentation of high resolution structural MR data. Other techniques that do not rely on structural MR data are available, such as deconvolution based approaches [40] and resolution recovery techniques incorporated into image reconstruction [41], although they generally cannot achieve full recovery of the resolution. Nevertheless, these techniques is worth investigating further as well because they can be applied in cases where only PET data are available. One approach we took to assess the stability and noise properties of reference region tracer uptake was based on an IDAIF technique using population based parent compound ratio for metabolites correction [30]. It should be pointed out that there is potentially group difference in tracer metabolism as well as inter individual variability which may introduce noise into the estimated tracer uptake. This can be investigated by performing actual metabolites measurement in a similar cohort. Nevertheless, the observation we obtained using this approach is consistent with SUV analysis. Additional investigation is also warranted on the feasibility of using an IDAIF approach for absolute quantification of amyloid burden in general.

## Conclusion

We examined several technical variations in the approach to obtain quantitative and semi-quantitative measurement of amyloid burden using PET imaging. PVC emerged as the strategy that most consistently improved statistical power for the detection of both longitudinal changes and across-group differences. This result accords with several previously reported studies [16,18,21]. Among several reference regions, core white matter provided the greatest sensitivity for the detection of longitudinal changes. However, the observed longitudinal change using white matter reference may be confounded by processes unrelated to amyloid burden and requires further investigation. We observed some advantages of acquiring full dynamic PET rather than shorter scans. However, given the countervailing costs, it remains unclear whether the advantages of full dynamic scanning outweigh the disadvantages. For the ADAD population with PiB imaging, utilizing brainstem as a reference region with PVC may be optimal for current interventional trials. Further investigation of technical issues in quantitative amyloid imaging in different study populations using different amyloid imaging tracers is warranted.

## References

[1] Holtzman DM, Morris JC, Goate AM Alzheimer's Disease: The Challenge of the Second Century. Science Translational Medicine 2011;3: 77sr71–77sr71. 10.1126/scitranslmed.3002369 PMC3130546 21471435

[2] Alzheimer's A 2014 Alzheimer's disease facts and figures. Alzheimers Dement 2014;10: e47–92. 24818261 10.1016/j.jalz.2014.02.001

[3] Bateman RJ, Xiong C, Benzinger TL, Fagan AM, Goate A, Fox NC, et al Clinical and biomarker changes in dominantly inherited Alzheimer's disease. N Engl J Med 2012;367: 795–804. 10.1056/NEJMoa1202753 22784036 PMC3474597

[4] Benzinger TL, Blazey T, Jack CR Jr., Koeppe RA, Su Y, Xiong C, et al Regional variability of imaging biomarkers in autosomal dominant Alzheimer's disease. Proc Natl Acad Sci U S A 2013;110: E4502–4509. 10.1073/pnas.1317918110 24194552 PMC3839740

[5] Aisen PS, Andrieu S, Sampaio C, Carrillo M, Khachaturian ZS, Dubois B, et al Report of the task force on designing clinical trials in early (predementia) AD. Neurology 2011;76: 280–286. 10.1212/WNL.0b013e318207b1b9 21178097 PMC3034393

[6] Aisen PS Alzheimer's disease therapeutic research: the path forward. Alzheimers Res Ther 2009;1: 2 10.1186/alzrt2 19674435 PMC2719107

[7] Schneider LS, Mangialasche F, Andreasen N, Feldman H, Giacobini E, Jones R, et al Clinical trials and late-stage drug development for Alzheimer's disease: an appraisal from 1984 to 2014. J Intern Med 2014;275: 251–283. 10.1111/joim.12191 24605808 PMC3956752

[8] Mills SM, Mallmann J, Santacruz AM, Fuqua A, Carril M, Aisen PS, et al Preclinical trials in autosomal dominant AD: implementation of the DIAN-TU trial. Rev Neurol (Paris) 2013;169: 737–743. 24016464 10.1016/j.neurol.2013.07.017 PMC3880800

[9] Sperling RA, Rentz DM, Johnson KA, Karlawish J, Donohue M, Salmon DP, et al The A4 study: stopping AD before symptoms begin? Sci Transl Med 2014;6: 228fs213. 10.1126/scitranslmed.3007941 PMC4049292 24648338

[10] Reiman EM, Langbaum JB, Fleisher AS, Caselli RJ, Chen K, Ayutyanont N, et al Alzheimer's Prevention Initiative: a plan to accelerate the evaluation of presymptomatic treatments. J Alzheimers Dis 2011;26 Suppl 3: 321–329. 10.3233/JAD-2011-0059 21971471 PMC3343739

[11] Mintun MA, Larossa GN, Sheline YI, Dence CS, Lee SY, Mach RH, et al [11C]PIB in a nondemented population: potential antecedent marker of Alzheimer disease. Neurology 2006;67: 446–452. 16894106 10.1212/01.wnl.0000228230.26044.a4

[12] Su Y, D'Angelo GM, Vlassenko AG, Zhou G, Snyder AZ, Marcus DS, et al Quantitative analysis of PiB-PET with FreeSurfer ROIs. PLoS One 2013;8: e73377 10.1371/journal.pone.0073377 24223109 PMC3819320

[13] Lippa CF, Saunders AM, Smith TW, Swearer JM, Drachman DA, Ghetti B, et al Familial and sporadic Alzheimer's disease: neuropathology cannot exclude a final common pathway. Neurology 1996;46: 406–412. 8614503 10.1212/wnl.46.2.406

[14] Mann DM, Pickering-Brown SM, Takeuchi A, Iwatsubo T Amyloid angiopathy and variability in amyloid beta deposition is determined by mutation position in presenilin-1-linked Alzheimer's disease. Am J Pathol 2001;158: 2165–2175. 11395394 10.1016/s0002-9440(10)64688-3 PMC1891993

[15] Edison P, Hinz R, Ramlackhansingh A, Thomas J, Gelosa G, Archer HA, et al Can target-to-pons ratio be used as a reliable method for the analysis of [(11)C]PIB brain scans? Neuroimage 2012;60: 1716–1723. 10.1016/j.neuroimage.2012.01.099 22306804

[16] Brendel M, Hogenauer M, Delker A, Sauerbeck J, Bartenstein P, Seibyl J, et al Improved longitudinal [(18)F]-AV45 amyloid PET by white matter reference and VOI-based partial volume effect correction. Neuroimage 2015;108: 450–459. 10.1016/j.neuroimage.2014.11.055 25482269

[17] Chen K, Roontiva A, Thiyyagura P, Lee W, Liu X, Ayutyanont N, et al Improved power for characterizing longitudinal amyloid-beta PET changes and evaluating amyloid-modifying treatments with a cerebral white matter reference region. J Nucl Med 2015;56: 560–566. 10.2967/jnumed.114.149732 25745091

[18] Su Y, Blazey TM, Snyder AZ, Raichle ME, Marcus DS, Ances BM, et al Partial volume correction in quantitative amyloid imaging. Neuroimage 2015;107: 55–64. 10.1016/j.neuroimage.2014.11.058 25485714 PMC4300252

[19] Soret M, Bacharach SL, Buvat I Partial-volume effect in PET tumor imaging. J Nucl Med 2007;48: 932–945. 17504879 10.2967/jnumed.106.035774

[20] Meltzer CC, Kinahan PE, Greer PJ, Nichols TE, Comtat C, Cantwell MN, et al Comparative evaluation of MR-based partial-volume correction schemes for PET. J Nucl Med 1999;40: 2053–2065. 10616886

[21] Thomas BA, Erlandsson K, Modat M, Thurfjell L, Vandenberghe R, Ourselin S, et al The importance of appropriate partial volume correction for PET quantification in Alzheimer's disease. Eur J Nucl Med Mol Imaging 2011;38: 1104–1119. 10.1007/s00259-011-1745-9 21336694

[22] Lopresti BJ, Klunk WE, Mathis CA, Hoge JA, Ziolko SK, Lu X, et al Simplified quantification of Pittsburgh Compound B amyloid imaging PET studies: a comparative analysis. J Nucl Med 2005;46: 1959–1972. 16330558

[23] Logan J, Fowler JS, Volkow ND, Wang GJ, Ding YS, Alexoff DL Distribution volume ratios without blood sampling from graphical analysis of PET data. Journal of Cerebral Blood Flow and Metabolism 1996;16: 834–840. 8784228 10.1097/00004647-199609000-00008

[24] Lammertsma AA, Hume SP Simplified reference tissue model for PET receptor studies. Neuroimage 1996;4: 153–158. 9345505 10.1006/nimg.1996.0066

[25] Landau SM, Fero A, Baker SL, Koeppe R, Mintun M, Chen K, et al Measurement of Longitudinal beta-Amyloid Change with 18F-Florbetapir PET and Standardized Uptake Value Ratios. J Nucl Med 2015;56: 567–574. 10.2967/jnumed.114.148981 25745095 PMC5313473

[26] Morris JC, Aisen PS, Bateman RJ, Benzinger TL, Cairns NJ, Fagan AM, et al Developing an international network for Alzheimer research: The Dominantly Inherited Alzheimer Network. Clin Investig (Lond) 2012;2: 975–984. 10.4155/cli.12.93 PMC3489185 23139856

[27] Goate A, Chartier-Harlin MC, Mullan M, Brown J, Crawford F, Fidani L, et al Segregation of a missense mutation in the amyloid precursor protein gene with familial Alzheimer's disease. Nature 1991;349: 704–706. 1671712 10.1038/349704a0

[28] Sherrington R, Rogaev EI, Liang Y, Rogaeva EA, Levesque G, Ikeda M, et al Cloning of a gene bearing missense mutations in early-onset familial Alzheimer's disease. Nature 1995;375: 754–760. 7596406 10.1038/375754a0

[29] Levy-Lahad E, Wasco W, Poorkaj P, Romano DM, Oshima J, Pettingell WH, et al Candidate gene for the chromosome 1 familial Alzheimer's disease locus. Science 1995;269: 973–977. 7638622 10.1126/science.7638622

[30] Su Y, Blazey TM, Snyder AZ, Raichle ME, Hornbeck RC, Aldea P, et al Quantitative amyloid imaging using image-derived arterial input function. PLoS One 2015;10: e0122920 10.1371/journal.pone.0122920 25849581 PMC4388540

[31] Morris JC The Clinical Dementia Rating (CDR): current version and scoring rules. Neurology 1993;43: 2412–2414. 10.1212/wnl.43.11.2412-a 8232972

[32] Pastor P, Roe CM, Villegas A, Bedoya G, Chakraverty S, Garcia G, et al Apolipoprotein Eepsilon4 modifies Alzheimer's disease onset in an E280A PS1 kindred. Ann Neurol 2003;54: 163–169. 12891668 10.1002/ana.10636

[33] Rousset OG, Collins DL, Rahmim A, Wong DF Design and implementation of an automated partial volume correction in PET: application to dopamine receptor quantification in the normal human striatum. J Nucl Med 2008;49: 1097–1106. 10.2967/jnumed.107.048330 18552147 PMC3104499

[34] Su Y, Arbelaez AM, Benzinger TL, Snyder AZ, Vlassenko AG, Mintun MA, et al Noninvasive estimation of the arterial input function in positron emission tomography imaging of cerebral blood flow. J Cereb Blood Flow Metab 2013;33: 115–121. 10.1038/jcbfm.2012.143 23072748 PMC3597366

[35] Logan J, Fowler JS, Volkow ND, Wolf AP, Dewey SL, Schlyer DJ, et al Graphical analysis of reversible radioligand binding from time-activity measurements applied to [N-11C-methyl]-(-)-cocaine PET studies in human subjects. J Cereb Blood Flow Metab 1990;10: 740–747. 2384545 10.1038/jcbfm.1990.127

[36] van Berckel BN, Ossenkoppele R, Tolboom N, Yaqub M, Foster-Dingley JC, Windhorst AD, et al Longitudinal amyloid imaging using 11C-PiB: methodologic considerations. J Nucl Med 2013;54: 1570–1576. 10.2967/jnumed.112.113654 23940304

[37] Joshi AD, Pontecorvo MJ, Clark CM, Carpenter AP, Jennings DL, Sadowsky CH, et al Performance characteristics of amyloid PET with florbetapir F 18 in patients with alzheimer's disease and cognitively normal subjects. J Nucl Med 2012;53: 378–384. 10.2967/jnumed.111.090340 22331215

[38] Bilgel M, Carass A, Resnick SM, Wong DF, Prince JL Deformation field correction for spatial normalization of PET images. Neuroimage 2015;119: 152–163. 10.1016/j.neuroimage.2015.06.063 26142272 PMC4564310

[39] Raniga P, Bourgeat P, Fripp J, Acosta O, Villemagne VL, Rowe C, et al Automated (11)C-PiB standardized uptake value ratio. Acad Radiol 2008;15: 1376–1389. 10.1016/j.acra.2008.07.006 18995189

[40] Tohka J, Reilhac A Deconvolution-based partial volume correction in Raclopride-PET and Monte Carlo comparison to MR-based method. Neuroimage 2008;39: 1570–1584. 18077187 10.1016/j.neuroimage.2007.10.038

[41] Panin VY, Kehren F, Michel C, Casey M Fully 3-D PET reconstruction with system matrix derived from point source measurements. IEEE Trans Med Imaging 2006;25: 907–921. 16827491 10.1109/tmi.2006.876171

