# Correction: Quantitative Amyloid Imaging in Autosomal Dominant Alzheimer’s Disease: Results from the DIAN Study Group

**DOI:** 10.1371/journal.pone.0163669

**Published:** 2016-09-20

**Authors:** Yi Su, Tyler M. Blazey, Christopher J. Owen, Jon J. Christensen, Karl Friedrichsen, Nelly Joseph-Mathurin, Qing Wang, Russ C. Hornbeck, Beau M. Ances, Abraham Z. Snyder, Lisa A. Cash, Robert A. Koeppe, William E. Klunk, Douglas Galasko, Adam M. Brickman, Eric McDade, John M. Ringman, Paul M. Thompson, Andrew J. Saykin, Bernardino Ghetti, Reisa A. Sperling, Keith A. Johnson, Stephen P. Salloway, Peter R. Schofield, Colin L. Masters, Victor L. Villemagne, Nick C. Fox, Stefan Förster, Kewei Chen, Eric M. Reiman, Chengjie Xiong, Daniel S. Marcus, Michael W. Weiner, John C. Morris, Randall J. Bateman, Tammie L. S. Benzinger, Dominantly Inherited Alzheimer Network

There are errors in the published article. Incorrect sample sizes were reported in Table 4 and Table 6. The intended sample size calculation was based on two-sided two-sample t-test to estimate the number of participants per arm needed to detect the specified reduction in amyloid accumulation rate due to treatment with 80% power and two-tailed type-I error of p = 0.05 in a 12-month placebo-controlled randomized clinical trial. The tables with corrected sample size values can be seen here.

**Table 4:** Longitudinal SUVR analysis for mean cortical regions in mutation carriers.

	MC_CER	MC_BS	MC_CW	MC_TW	MCRSF_CER	MCRSF_BS	MCRSF_CW	MCRSF_TW
**Baseline**	1.73±0.58	1.15±0.35	1.08±0.31	0.99±0.14	2.54±1.28	1.43±0.63	1.53±0.73	1.43±0.56
**follow-up**	1.76±0.60	1.17±0.35	1.12±0.32	1.00±0.14	2.65±1.36	1.48±0.64	1.64±0.80	1.49±0.57
**delta**	0.03±0.11	0.01±0.07	0.04±0.07	0.01±0.03	0.11±0.26	0.05±0.10	0.11±0.17	0.06±0.11
**delta%**	1.63±7.04	1.51±6.30†	3.34±5.88	1.29±3.54	4.19±11.07*‡	4.08±9.86*‡	7.03±11.27*	5.05±9.82*
**p (follow-up vs. Baseline)**	7.22E-02	1.39E-01	1.70E-05	8.12E-03	8.78E-04	6.25E-04	7.46E-06	1.14E-04
**Rate**	0.01±0.08	0.00±0.04	0.02±0.04	0.01±0.02	0.07±0.18	0.02±0.06	0.07±0.13	0.04±0.07
**Effect Size**	0.15	0.07	0.55	0.38	0.38	0.38	0.51	0.50
**sample size (25% reduction in Rate)**	11425	48016	819	1744	1712	1701	956	1001
**sample size (50% reduction in rate)**	2857	12005	206	437	429	426	240	251

MC_CER = mean cortical region SUVR using cerebellar cortex as reference; MC_BS = mean cortical region SUVR using brainstem as reference; MC_CW = mean cortical region SUVR using core white matter as reference; MC_TW = mean cortical region SUVR using total white matter as reference; MCRSF_CER = mean cortical region SUVR using cerebellar cortex as reference with RSF partial volume correction; MCRSF_BS = mean cortical region SUVR using brainstem as reference with RSF partial volume correction; MCRSF_CW = mean cortical region SUVR using core white matter as reference with RSF partial volume correction; MCRSF_TW = mean cortical region SUVR using total white matter as reference with RSF partial volume correction; delta = change in SUVR from baseline to follow-up; delta% = percent change in SUVR from baseline to follow-up; p is the strength of the difference between follow-up and baseline SUVRs based on a paired t-test; Rate = the annual rate of SUVR change; sample size is the estimated number of participants per arm needed to detect a 25% or a 50% reduction in amyloid accumulation rate due to treatment with 80% power and a two-tailed type-I error of p = 0.05 in a 12-month placebo-controlled randomized clinical trial.

*percent change in MCSUVR significantly greater with PVC than without (p<0.0005)

†percent change in MCSUVR significantly smaller than CW referencing (p<0.01)

‡percent change in MCSUVR with PVC significantly smaller than CW referencing (p<0.05)

**Table 6:** Mean cortical measurement for longitudinal cohort participants with full dynamic PiB.

	MC_CER	MC_BS	MC_CW	MC_TW	MCRSF_CER	MCRRSF_BS	MCRRSF_CW	MCRRSF_TW	MCBP	MCBPRSF
**Baseline**	1.83±0.59	1.18±0.35	1.12±0.31	1.02±0.16	2.80±1.33	1.50±0.64	1.60±0.71	1.54±0.62	0.62±0.45	1.33±0.93
**follow-up**	1.89±0.58	1.22±0.35	1.16±0.32	1.03±0.15	2.93±1.33	1.58±0.66	1.71±0.75	1.60±0.62	0.67±0.45	1.43±0.95
**delta**	0.05±0.13	0.04±0.06	0.04±0.05	0.01±0.04	0.14±0.29	0.09±0.09	0.11±0.12	0.06±0.11	0.05±0.10	0.10±0.22
**p (follow-up vs. Baseline)**	6.34E-02	1.98E-03	4.47E-04	8.39E-02	3.33E-02	2.02E-04	1.68E-04	1.19E-02	2.91E-02	3.84E-02
**Rate**	0.02±0.09	0.02±0.03	0.02±0.03	0.00±0.02	0.05±0.17	0.04±0.05	0.05±0.06	0.03±0.05	0.02±0.05	0.04±0.11
**Effect Size**	0.23	0.62	0.71	0.25	0.33	0.85	0.82	0.49	0.33	0.34
**sample size (25% reduction in Rate)**	4570	662	499	4072	2339	350	373	1034	2350	2192
**sample size (50% reduction in rate)**	1143	167	126	1019	586	89	94	260	589	549

MC_CER = mean cortical region SUVR using cerebellar cortex as reference; MC_BS = mean cortical region SUVR using brainstem as reference; MC_CW = mean cortical region SUVR using core white matter as reference; MC_TW = mean cortical region SUVR using total white matter as reference; MCRSF_CER = mean cortical region SUVR using cerebellar cortex as reference with RSF partial volume correction; MCRSF_BS = mean cortical region SUVR using brainstem as reference with RSF partial volume correction; MCRSF_CW = mean cortical region SUVR using core white matter as reference with RSF partial volume correction; MCRSF_TW = mean cortical region SUVR using total white matter as reference with RSF partial volume correction; MCBP = mean cortical binding potential; MCBPRSF = mean cortical binding potential with RSF partial volume correction; delta = change in SUVR from baseline to follow-up; p is the strength of the difference between follow-up and baseline SUVRs based on a paired t-test; Rate = the annual rate of SUVR change; sample size is the estimated number of participants per arm needed to detect a 25% or a 50% reduction in amyloid accumulation rate due to treatment with 80% power and a two-tailed type-I error of p = 0.05 in a 12-month placebo-controlled randomized clinical trial.
